# Method for Translation and Rotation Decoupling of Test Mass in Full-Maglev Vertical Superconducting Gravity Instruments

**DOI:** 10.3390/s20195527

**Published:** 2020-09-27

**Authors:** Lulu Wang, Daiyong Chen, Xikai Liu, Liang Chen, Xiangdong Liu

**Affiliations:** MOE Key Laboratory of Fundamental Physical Quantities Measurement & Hubei Key Laboratory of Gravitation and Quantum Physics, School of Physics, Huazhong University of Science and Technology, Wuhan 430074, China; d201880048@hust.edu.cn (L.W.); chendai@hust.edu.cn (D.C.); liangchen@hust.edu.cn (L.C.); liuxd@hust.edu.cn (X.L.)

**Keywords:** superconducting gravity instruments, superconducting circuit design, motion decoupling

## Abstract

For full-maglev vertical superconducting gravity instruments, displacement control in the non-sensitive axis is a key technique to suppress cross-coupling noise in a dynamic environment. Motion decoupling of the test mass is crucial for the control design. In practice, when levitated, the test mass is always in tilt, and unknown parameters will be introduced to the scale factors of displacement detection, which makes motion decoupling work extremely difficult. This paper proposes a method for decoupling the translation and rotation of the test mass in the non-sensitive axis for full-maglev vertical superconducting gravity instruments. In the method, superconducting circuits at low temperature and adjustable gain amplifiers at room temperature are combined to measure the difference between the scale factors caused by the tilt of the test mass. With the measured difference of the scale factors, the translation and rotation are decoupled according to the theoretical model. This method was verified with a test of a home-made full-maglev vertical superconducting accelerometer in which the translation and rotation were decoupled.

## 1. Introduction

Full-maglev vertical superconducting gravity instruments (FM-VSGIs) possess numerous advantages compared with traditional instruments thanks to the lack of mechanical connection between the test mass and the base of the instrument. Due to benefits such as low drift and high sensitivity [1,2,3], these instruments have a broad range of applications in geodynamic studies, natural disaster monitoring, and resource exploration [4,5,6,7]. In FM-VSGIs, superconducting levitating coils are used to levitate the test mass in the sensitive axis (the vertical direction), and side-wall superconducting coils are used to provide the needed stiffness to limit the motion of the test mass in the non-sensitive axes (the horizontal direction) [8,9]. The motion of the test mass has multiple degrees of freedom. Due to factors such as mismatch of coils and installation error, the motions of the test mass in the non-sensitive axes couple to the desired signals in the sensitive axis, causing higher measurement noise. To suppress the cross-coupling between the sensitive and non-sensitive axes, a number of approaches have been presented.

In the superconducting gravity instruments developed by the University of Maryland and the University of Western Australia [10,11,12], the test mass is mechanically connected to the base. Thus, significant stiffness is provided in the non-sensitive axes, so the displacements of the test mass in the non-sensitive axes are small. The coupling coefficients can be measured accurately and, according to the noise model, cross-coupling noise is reduced by data processing. Researchers have successfully suppressed cross-coupling noise in this manner [13,14]. However, in FM-VSGIs, which are limited by the critical current of the superconducting coils, because the stiffness cannot be as high as that of the mechanically connected one, the displacements of the test mass in the non-sensitive axes are much larger, especially in a dynamic environment [10]. In this situation, the attitude of the test mass changes continually with a large margin. Thus, the measurement accuracy of the coupling coefficients is significantly limited and cross-coupling noise cannot be sufficiently reduced.

In FM-VSGIs, the test mass must be controlled to make it relatively static to the base, then the coupling coefficients can be measured accurately and a noise reduction method can be used. One key step in the control design is the motion decoupling of the test mass [15,16]. In practice, the test mass is always tilted when levitated, and the amplitude and direction of the tilt are hard to predict. As a result, the parameters of the superconducting coils are unknown, because these parameters are directly determined by the distance between the test mass and the coils. Motion decoupling cannot be carried out because of these unknown parameters even if the displacements are detected [17]. In the FM-VSGI developed by the ARKEX Company in the United Kingdom [18], to suppress cross-coupling noise, the researchers designed superconducting circuits for adjusting the sensitive axis of the test mass. However, this approach does not allow for the suppression of cross-coupling noise in a dynamic environment, because the displacement of the test mass must be first controlled, motion decoupling of the test mass is essential for control. In Ref. [19], J. W. Parke developed a “si*x*-axis” superconducting accelerometer. A complex test mass with 24 intersecting surfaces was used and 24 superconducting coils were utilized to act with the intersecting surfaces. In each axis, at least four superconducting coils were properly selected and superconducting circuits were designed to make the sensing circuit only sensitive to one motion degree of freedom. The motion of the test mass can be decoupled, and the decoupling efficiency depends mainly on the matching of the coils. Because this method of motion decoupling uses a large number of superconducting coils, the assembly of the instrument is extremely difficult and its reliability may be uncertain. How to deal with the unknown parameters introduced by the tilt of the test mass has not been reported in the literature.

This paper proposes a practical and simple method for motion decoupling. In the proposed approach, adjustable gain amplifiers at room temperature combining with the superconducting quantum interference devices (SQUIDs) are utilized to determine the difference between the unknown parameters introduced by the tilt of the test mass. This difference is artificially compensated for the output of the SQUIDs and decoupling of translation and rotation are then carried out by data processing according to a new motion model that takes account of the tilt of the test mass. The method was verified with a test of a home-made full-maglev vertical superconducting accelerometer (FM-VSA). The results showed that translation and rotation in the non-sensitive axis were decoupled successfully.

The rest of the paper is organized as follows: In Section 2, a theoretical analysis of the decoupling method with a brief introduction of the FM-VSA is presented; In Section 3, the design and results of the experiment are provided and discussed; Section 4 features the conclusions.

## 2. Theoretical Analysis

### 2.1. Process of the Decoupling Method

**Figure 1:**
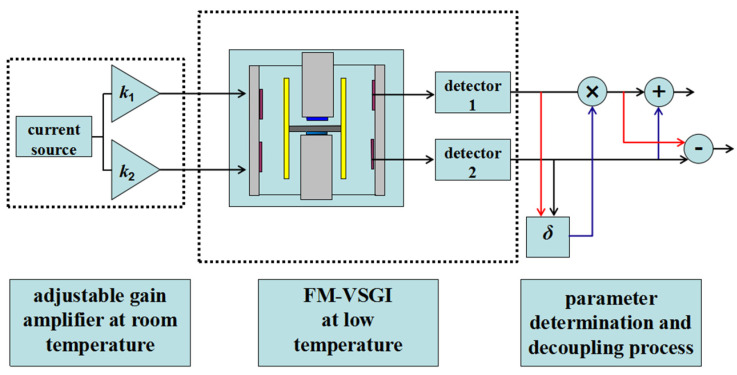
Schematic diagram of the decoupling method.

The basic process of the decoupling method is provided in Figure 1. The experimental apparatus used in the method contains the current source and adjustable gain amplifiers at room temperature, the FM-VSGI at low temperature and the parameter determination and decoupling system. In the proposed method, the current source and adjustable gain amplifiers at room temperature combining with the superconducting circuit are utilized to produce the pure translational force to vibrate the test mass in the non-sensitive axis. Two superconducting quantum interference devices (SQUIDs) are integrated to detect the displacement of the test mass. When pure translational acceleration is applied to the test mass, the difference between the unknown parameters introduced by the tilt of the test mass can be obtained from the output of the two SQUIDs. This difference is artificially multiplied by one of the outputs of the SQUIDs, and decoupling of translation and rotation can be then carried out by directly adding and subtracting the output of the SQUIDs. The principle of the method will be introduced in detail in the next section.

### 2.2. The Basic Structure of the FM-VSA

**Figure 2:**
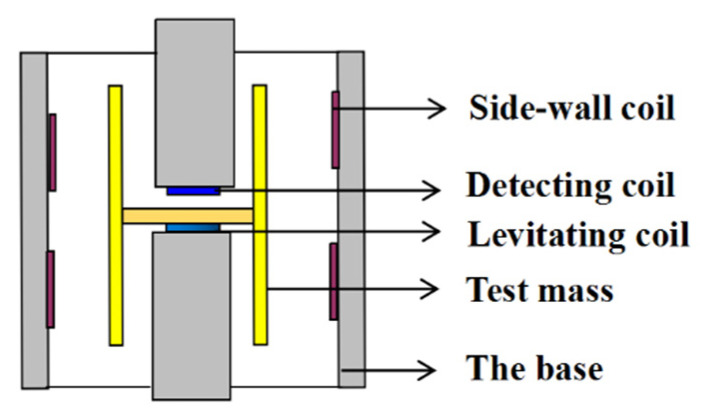
Schematic profile of the vertical superconducting accelerometer.

The basic structure of the FM-VSA is shown in Figure 2. The test mass is in the form of an axisymmetrically hollow cylinder with a division plate inside. Two types of superconducting coils are used in the instrument: planar coils and side-wall coils [8]. The planar coils include the levitating coil and detecting coil, and are used to levitate the test mass and detect the displacement of the test mass in the sensitive axis. The side-wall coils are used to provide the needed stiffness for the motion in the non-sensitive axes; for each non-sensitive axis, four side-wall coils are distributed symmetrically [8].

According to the Meissner effect of the superconductor, when persistent currents are injected into these coils, the test mass will be levitated, and spring oscillators in both the sensitive and non-sensitive axes are formed. The motion of the test mass has five degrees of freedom: translation and rotation in the two non-sensitive axes, and translation in the sensitive axis. The displacements of the test mass vary when the platform acceleration or gravity changes. The displacements are then detected by superconducting circuits and superconducting quantum interference devices (SQUIDs). SQUIDs are ultra-high sensitive devices integrated by the Josephson junctions and the read-out circuits [20], the sensitivity of SQUIDs is at the level of μΦ_0_/Hz^1/2^, where 1 Φ_0_ = 2.06 × 10^−15^ Wb. As a benefit of the SQUIDs, the sensitivity of the FM-VSA can be lower than 10^−9^ g/Hz^1/2^ [9].

In Ref. [8], X. Liu et al. introduced the structure and working principle of a homemade FM-VSA, and analyzed the motion of the test mass in the sensitive axis with a detailed calculation of the superconducting circuits. Ref. [8] provides further details. Here, we analyze the motion model of the test mass in the non-sensitive axis.

### 2.3. Theoretical Model

**Figure 3:**
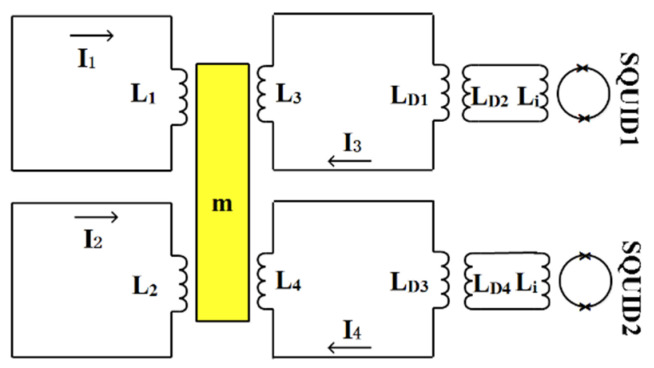
Superconducting circuits in the non-sensitive axis.

In the non-sensitive axis, we used the side-wall coils to design the superconducting circuits for displacement detection [8]. The structure of the superconducting circuits in the non-sensitive axis is shown in Figure 3.

*L*_1,_*L*_2_, *L*_3_, and *L*_4_ are the four side-wall coils, and each coil is connected to one circuit. At the beginning of the test, the same persistent current I_0_ is injected into the four circuits, and magnetic forces between the coils and the test mass are produced. The test mass is then held in an equilibrium position. Supposing the test mass has a leftward translational displacement *x* and rightward rotational angle *θ* about the center, for a small angle, the displacement between the coil and test mass can be expressed as *lθ*, where *l* is the force arm. According to the Meissner effect, the inductance of the four coils can be written as [8]:

(1) $$\left\{ \begin{array}{l} L_{1}=L_{10}\left(1+\lambda_{1}\left(-x+l\theta \right)\right)\\ L_{2}=L_{20}\left(1+\lambda_{2}\left(-x-l\theta \right)\right)\\ L_{3}=L_{30}\left(1+\lambda_{3}\left(x-l\theta \right)\right)\\ L_{4}=L_{40}\left(1+\lambda_{4}\left(x+l\theta \right)\right) \end{array}\right.$$

Here, *L*_10_, *L*_20_, *L*_30_, and *L*_40_ are the inductance of the four coils when the test mass is in the equilibrium position. *λ*_1_, *λ*_2_, *λ*_3_, and *λ*_4_ are the four coefficients of each coil related to the displacement. Then, according to the conservation of magnetic flux of the superconducting circuits [9]:

(2) $$\left\{ \begin{array}{l} L_{1}I_{1}=L_{10}I_{0}\\ L_{2}I_{2}=L_{20}I_{0}\\ \left(L_{3}+L_{D1}-\frac{M_{D1}{}^{2}}{L_{D2}+L_{i}}\right)I_{3}=\left(L_{30}+L_{D1}-\frac{M_{D1}{}^{2}}{L_{D2}+L_{i}}\right)I_{0}\\ \left(L_{4}+L_{D3}-\frac{M_{D2}{}^{2}}{L_{D4}+L_{i}}\right)I_{4}=\left(L_{40}+L_{D3}-\frac{M_{D2}{}^{2}}{L_{D4}+L_{i}}\right)I_{0} \end{array}\right.$$

Here, *L*_D1_, *L*_D2_, and *M*_D1_ are primary, secondary, and mutual inductance of the detecting transformer connected to SQUID1, respectively; *L*_D3_, *L*_D4_, and *M*_D2_ are primary, secondary, and mutual inductance of the detecting transformer connected to SQUID2, respectively; *L*_i_ is the inductance of the SQUID input coil.

The varying current in the circuits can be derived as:

(3) $$\left\{ \begin{array}{l} I_{1}=I_{0}\left(1-\lambda_{1}\left(-x+l\theta \right)\right)\\ I_{2}=I_{0}\left(1-\lambda_{2}\left(-x-l\theta \right)\right)\\ I_{3}=I_{0}\left(1-\frac{L_{30}\lambda_{3}}{L_{30}+L_{D1}-\frac{M_{D1}{}^{2}}{L_{D2}+L_{i}}}\left(x-l\theta \right)\right)\\ I_{4}=I_{0}\left(1-\frac{L_{40}\lambda_{4}}{L_{40}+L_{D3}-\frac{M_{D2}{}^{2}}{L_{D4}+L_{i}}}\left(x+l\theta \right)\right) \end{array}\right.$$

and the magnetic forces produced by the coils as:

(4) $$\left\{ \begin{array}{l} F_{1}=\frac{1}{2}\lambda_{1}I_{0}{}^{2}\left(1-2\lambda_{1}\left(-x+l\theta \right)\right)\\ F_{2}=\frac{1}{2}\lambda_{2}I_{0}{}^{2}\left(1-2\lambda_{2}\left(-x-l\theta \right)\right)\\ F_{3}=\frac{1}{2}\lambda_{3}I_{0}{}^{2}\left(1-\frac{2L_{30}\lambda_{3}}{L_{30}+L_{D1}-\frac{M_{D1}{}^{2}}{L_{D2}+L_{i}}}\left(x-l\theta \right)\right)\\ F_{4}=\frac{1}{2}\lambda_{4}I_{0}{}^{2}\left(1-\frac{2L_{40}\lambda_{4}}{L_{40}+L_{D3}-\frac{M_{D2}{}^{2}}{L_{D4}+L_{i}}}\left(x+l\theta \right)\right) \end{array}\right.$$

For the translation, forces acting on the test mass include the external translational force, the damping force and the electromagnetic forces produced by the coils. According to Newton’s second law, the dynamic equation of the test mass is:

(5) $$m\;\overset{..}{x}+c\;\dot{x}+\left(F_{1}+F_{2}+F_{3}+F_{4}\right)=ma_{//}$$

where *x* is the displacement of the test mass; *m* is the mass of the test mass; *c* is the damping coefficient; *a*_//_ is the external translational acceleration.

Combining Equations (4) and (5), using Fourier transform, we can obtain the relationship between the translational displacement *x* and the external acceleration *a*_//_:

(6) $$x\left(\omega \right)=\frac{ma_{//}}{-m\omega^{2}+jc_{//}\omega +K_{//}}$$

Here, *K*_//_ is the stiffness of translation mode, and is defined as:

(7) $$K_{//}=\frac{\partial \left(F_{1}+F_{2}+F_{3}+F_{4}\right)}{\partial x}=\left(\lambda_{1}{}^{2}+\lambda_{2}{}^{2}+\frac{L_{30}\lambda_{3}{}^{2}}{L_{30}+L_{D1}-\frac{M_{D1}{}^{2}}{L_{D2}+L_{i}}}+\frac{L_{40}\lambda_{4}{}^{2}}{L_{40}+L_{D3}-\frac{M_{D2}{}^{2}}{L_{D4}+L_{i}}}\right)I_{0}{}^{2}$$

For the rotation, the dynamic equation of the test mass is:

(8) $$I_{r}\;\overset{..}{\theta }+c_{r}\;\dot{\theta }+\left(-F_{1}+F_{2}+F_{3}-F_{4}\right)=I_{r}a_{r}$$

Here, *I*_r_ is the moment of inertia of the test mass; *c*_r_ is the damping coefficient of the rotational spring oscillator; *a*_r_ is the external rotational angular acceleration.

The relationship between the rotational angle *θ* and the external rotational acceleration *a*_r_ is:

(9) $$\theta \left(\omega \right)=\frac{I_{r}a_{r}}{-I_{r}\omega^{2}+jc_{r}\omega +K_{r}}$$

Here, *K*_r_ is the stiffness of rotation mode, and is defined as:

(10) $$K_{r}=\frac{\partial \left(-F_{1}+F_{2}+F_{3}-F_{4}\right)}{\partial \theta }=\left(\lambda_{1}{}^{2}+\lambda_{2}{}^{2}+\frac{L_{30}\lambda_{3}{}^{2}}{L_{30}+L_{D1}-\frac{M_{D1}{}^{2}}{L_{D2}+L_{i}}}+\frac{L_{40}\lambda_{4}{}^{2}}{L_{40}+L_{D3}-\frac{M_{D2}{}^{2}}{L_{D4}+L_{i}}}\right)I_{0}{}^{2}l$$

Superconducting transformers and SQUID are used to detect the attitude changes of the test mass, as shown in Figure 3. The currents flowing into the input coils of the SQUID are:

(11) $$\left\{ \begin{array}{l} i_{SQ1}=\frac{M_{D1}}{L_{D2}+L_{i}}I_{3}=K_{1}\left(x-l\theta \right)\\ i_{SQ2}=\frac{M_{D2}}{L_{D4}+L_{i}}I_{4}=K_{2}\left(x+l\theta \right) \end{array}\right.$$

where

(12) $$\left\{ \begin{array}{l} K_{1}=\frac{M_{D1}I_{0}}{L_{D2}+L_{i}}\frac{L_{30}\lambda_{3}}{L_{30}+L_{D1}-\frac{M_{D1}{}^{2}}{L_{D2}+L_{i}}}\\ K_{2}=\frac{M_{D1}I_{0}}{L_{D4}+L_{i}}\frac{L_{40}\lambda_{4}}{L_{40}+L_{D3}-\frac{M_{D2}{}^{2}}{L_{D4}+L_{i}}} \end{array}\right.$$

are the two scale factors of the displacements to the currents detected by the SQUID.

Based on Equation (11), the translational displacement *x* and rotational angle *θ* can be calculated from the detected currents *i*_SQ1_ and *i*_SQ2_:

(13) $$\left\{ \begin{array}{l} x=\frac{i_{SQ1}}{2K_{1}}+\frac{i_{SQ2}}{2K_{2}}\\ \theta =-\frac{i_{SQ1}}{2lK_{1}}+\frac{i_{SQ2}}{2lK_{2}} \end{array}\right.$$

Because of the tilt of the test mass, *λ*_3_ and *λ*_4_ in Equation (12) are not equal and are unknown. Thus, *i*_SQ1_ and *i*_SQ2_ are measured by the SQUID, and *x* and *θ* cannot be decoupled because *K*_1_ and *K*_2_ are determined by *λ*_3_ and *λ*_4_.

However, when *δ* = *K*_1_/*K*_2_ is known, one can multiply the measured *i*_SQ1_ by *δ* through data processing. Then, Equation (13) becomes:

(14) $$\left\{ \begin{array}{l} x=\frac{\delta \;i_{SQ1}}{2K_{1}}+\frac{\;i_{SQ2}}{2K_{2}}=\frac{1}{2K_{2}}\left(i_{SQ1}+i_{SQ2}\right)\\ \theta =-\frac{\delta \;i_{SQ1}}{2lK_{1}}+\frac{i_{SQ2}}{2lK_{2}}=\frac{1}{2lK_{2}}\left(-i_{SQ1}+i_{SQ2}\right) \end{array}\right.$$

Now, *i*_SQ1_ and *i*_SQ2_ can be directly added and subtracted to obtain *x* and *θ*, and the translation and rotation are decoupled. Notably, the scale factors of the decoupled *x* and *θ* are not the actual factors. For control design, this shortcoming can be compensated for by adjusting the PID parameters. In addition, the scale factors can be determined by applying translational vibrating acceleration to the instrument if needed.

In the method, we determine *δ* by applying pure translational force to vibrate the test mass. When translational force is applied, the displacement of the test mass only contains *x*, and the detected currents *i*_SQ1_ and *i*_SQ2_ of the two SQUIDs in Equation (11) become:

(15) $$\left\{ \begin{array}{l} i_{SQ1}=K_{1}x\\ i_{SQ2}=K_{2}x \end{array}\right.$$

*δ* can be calculated from *i*_SQ1_ and *i*_SQ2_:

(16) $$\delta =\frac{i_{SQ1}}{i_{SQ2}}$$

**Figure 4:**
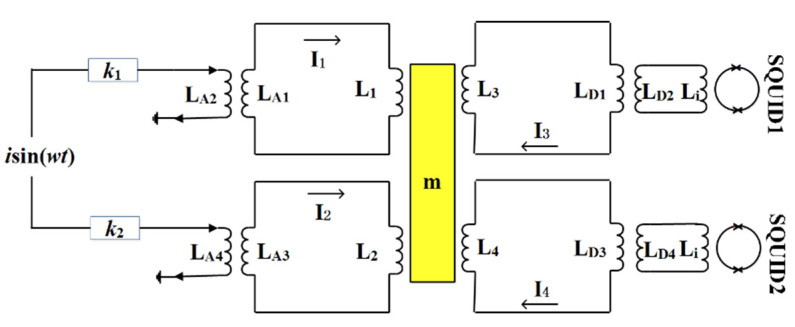
Circuits for the measurement of *δ*. Adjustable gain amplifiers are used.

The key of the method lies in how to provide a pure translational acceleration to vibrate the test mass. The superconducting circuit shown in Figure 4 was designed.

Ideally, the sensitive axis of the test mass is in the vertical direction, parameters of the side-wall coils are equal to each other, when the same current flows through L_1_ and L_2_, equal forces will be produced, and the forces can be equivalent to a translational force acting on the test mass. However, the test mass is always tilted in practice, parameters of L_1_ and L_2_ are not equal. In this case, in order to provide a translational force, adjustable variables must be introduced into the system to compensate for the difference between L_1_ and L_2_. In our designed circuit, adjustable gain amplifiers at room temperature are used to achieve this goal.

The actuating method was described in Ref. [21], a brief calculation of the circuit is provided here. When a sinusoidal current is coupled into the superconducting loops through the adjustable gain amplifiers and the transformers, the total currents I_1_ and I_2_ can be calculated from the flux conservation of the two loops. The results are:

(17) $$\left\{ \begin{array}{l} I_{1}=I_{0}+\frac{k_{1}M_{A1}}{L_{1}+L_{A1}}i\sin\left(\omega t\right)\\ I_{2}=I_{0}+\frac{k_{2}M_{A2}}{L_{2}+L_{A3}}i\sin\left(\omega t\right) \end{array}\right.$$

Where L_A1_, L_A3_, M_A1_, and M_A2_ are the primary and mutual inductance of the actuating transformers; *k*_1_ and *k*_2_ are the adjustable gains of the amplifiers.

The actuating forces produced by the two coils are calculated from Equation (17):

(18) $$\left\{ \begin{array}{l} F_{1}=\frac{1}{2}\lambda_{1}I_{1}{}^{2}\approx \frac{1}{2}\lambda_{1}I_{0}{}^{2}+\lambda_{1}\frac{k_{1}M_{A1}}{\left(L_{1}+L_{A1}\right)}I_{0}i\sin\left(\omega t\right)\\ F_{2}=\frac{1}{2}\lambda_{2}I_{2}{}^{2}\approx \frac{1}{2}\lambda_{2}I_{0}{}^{2}+\lambda_{2}\frac{k_{2}M_{A2}}{\left(L_{2}+L_{A3}\right)}I_{0}i\sin\left(\omega t\right) \end{array}\right.$$

In Equation (18), mismatches exist between the parameters of the transformers and *λ*_1_ and *λ*_2_. To provide the pure translational force, the second terms on the right side of Equation (18) should satisfy:

(19) $$\lambda_{1}\frac{k_{1}M_{1}}{L_{1}+L_{A1}}=\lambda_{2}\frac{k_{2}M_{2}}{L_{2}+L_{A3}}$$

By adjusting *k*_1_ and *k*_2_, the goal of producing pure translational force can be achieved. *δ* will then be determined according to Equation (16) and decoupling of the translation and rotation can be carried out by data processing according to Equation (14).

## 3. Experimental Test

The decoupling method was verified in the test of a homemade FM-VSA. By applying translational acceleration to vibrate the test mass, we measured the amplitude–frequency response functions and determined the difference between *K*_1_ and *K*_2_. The translation and rotation of the test mass were then decoupled successfully. The experiment and results are discussed in this section.

The FM-VSA was installed in a vacuum chamber of a helium Dewar. After persistent currents were injected into the superconducting circuits, original adjustments were made to ensure that the test mass was levitated and moved freely. A sinusoidal current was then injected into the circuit by a signal generator to vibrate the test mass, and the responses of the SQUID were recorded and compared. In the measurement, the amplitude of the AC current was fixed, and the frequency ranged from 1 to 30 Hz.

**Figure 5:**
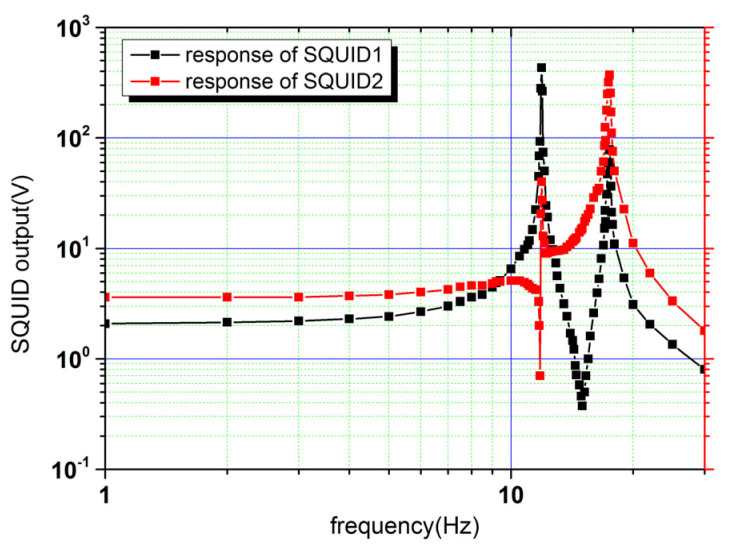
Original amplitude–frequency response functions of the two superconducting circuits and superconducting quantum interference devices (SQUIDs) to the applied actuating accelerations.

In the measurements, the amplitude of the sinusoidal current was set as 10 mA. The current was injected into the superconducting circuits in Figure 4 to vibrate the test mass, and the outputs of the two SQUIDs were summarized at each frequency point to obtain the amplitude–frequency response functions. Before adjusting the gains of the amplifiers, *k*_1_ was set to be equal to *k*_2_. Due to the tilt of the test mass, the second items on the right side of Equation (18) are not equal. Furthermore, both translational and rotational accelerations are produced and their amplitudes are unknown. Thus, the amplitude–frequency response functions were represented by the output voltages of the SQUID. The measured result is shown in Figure 5.

Two resonance peaks and two valleys appear in both of the curves, which is consistent with the theoretical model. Two resonance peaks represent the resonance frequencies of the rotation and translation. At the frequency points of the two valleys, the translational displacement *x* and the equivalent rotational displacement *lθ* canceled each other, representing the zero points of Equation (11).

**Figure 6:**
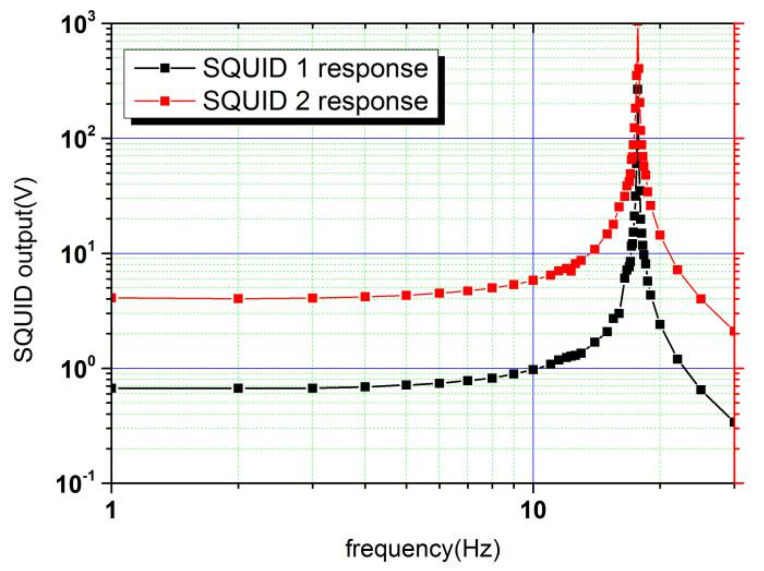
Responses of the two SQUIDs to the applied translational actuating acceleration when *k*_1_ and *k*_2_ were adjusted.

*k*_1_ and *k*_2_ were then adjusted iteratively, and the amplitude–frequency response functions of the two SQUIDs were compared until pure translation responses were achieved. The final result is shown in Figure 6.

Here, only the resonance frequency of translation appeared in the responses, showing that pure translation was achieved. Now, *δ* can be calculated as:

(20) $$\delta =\frac{i_{SQ1}}{i_{SQ2}}=0.16$$

**Figure 7:**
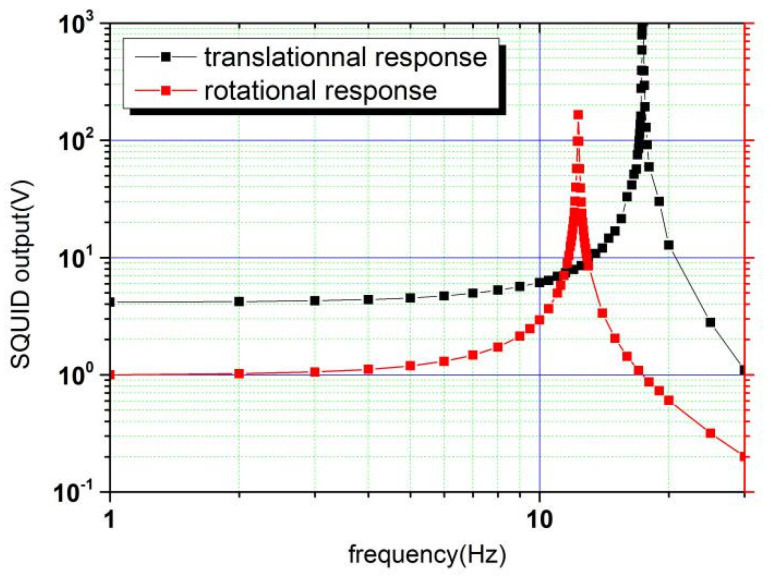
Decoupling of the translation and rotation by the proposed method.

Using this ratio and according to Equation (14), the translation and rotation of the test mass were decoupled based on the measured data in Figure 5. The result is shown in Figure 7.

The red line only contains the rotational resonance frequency and the black line only contains the translational resonance frequency. Thus, the translation and rotation of the test mass were decoupled. The proposed and verified method may make up for the deficiency of the research mentioned in Ref. [18]. Notably, in our method, amplifiers with thermal noise at room temperature are used. The amplifiers are only used to determine *δ.* Once this is achieved, the connections between the amplifiers and the superconducting circuits are broken, and no external thermal noise is introduced into the measurement of the FM-VSGI.

The successful application of the decoupling method will yield two advantages for the development of FM-VSGIs. First, the method can be used to decouple motion systems with multiple degrees of freedom as a combination of numerous independent motion systems with a single degree of freedom, thus simplifying the design of the control system. After displacement control is achieved, cross-coupling noise can be suppressed significantly, and the sensitivity of the FM-VSGI will be improved accordingly. Second, the approach makes it possible to develop multiple-degrees-of-freedom full-maglev superconducting gravity instruments in which only one simply shaped test mass is levitated. When the motions of the test mass in both the non-sensitive axes are decoupled and calibrated, the FM-VSGI has the ability to measure the acceleration signals in five degrees of freedom, namely three translational accelerations in both the vertical and horizontal directions, and two rotational accelerations in the horizontal direction.

## 4. Conclusions

A method for the decoupling of the translation and rotation of the test mass in an FM-VSGI was introduced. Adjustable gain amplifiers combined with superconducting circuits were used to determine the difference of the scale factors caused by the tilt of the test mass. This method was verified in a test of an FM-VSA. Results showed that the translation and rotation were decoupled successfully. This work lays a foundation for control system design and may assist in the development of multiple-degree-of-freedom superconducting gravity instruments.
